# Molecular interplay between T-Antigen and splicing factor, arginine/serine-rich 1 (SRSF1) controls JC virus gene expression in glial cells

**DOI:** 10.1186/s12985-015-0426-x

**Published:** 2015-11-24

**Authors:** Michael Craigie, Patrick Regan, Yolanda-Lopez Otalora, Ilker Kudret Sariyer

**Affiliations:** Department of Neuroscience, Center for Neurovirology, Temple University Lewis Katz School of Medicine, 3500 North Broad Street, 7th Floor, Philadelphia, PA 19140 USA

## Abstract

**Background:**

Human polyomavirus JCV is the etiologic agent of progressive multifocal leukoencephalopathy (PML), a fatal demyelinating disease characterized by lytic infection of glial cells in the central nervous system. PML is seen primarily in immunosuppressed patients and is mainly classified as an AIDS-defining disease. In addition to structural capsid proteins, JCV encodes multiple regulatory proteins, including T-antigen and agnoprotein, which are required for functional lytic infection. Previous studies have suggested that molecular interaction between viral proteins and host factors play an important role in reactivation of JCV and progression of the viral life cycle in glial cells. Recently, serine/arginine rich splicing factor 1 (SRSF1), a cellular alternative splicing factor, was identified as a strong negative regulator of JCV in glial cells. SRSF1 inhibits JCV gene expression and viral replication by directly interacting with viral promoter sequences. Here, we have investigated possible impact of JCV regulatory proteins, T-antigen and agnoprotein, on SRSF1-mediated suppression of JCV gene expression in glial cells.

**Results:**

Reporter gene analysis has suggested that T-antigen rescues viral transcriptional suppression mediated by SRSF1. Further analyses have revealed that T-antigen promotes viral gene expression by suppressing SRSF1 gene transcription in glial cells. A subsequent ChIP analysis revealed that T-antigen associates with the promoter region of SRSF1 to induce the transcriptional suppression.

**Conclusions:**

These findings have revealed a molecular interplay between cellular SRSF1 and viral T-antigen in controlling JCV gene expression, and may suggest a novel mechanism of JCV reactivation in patients who are at risk of developing PML.

## Introduction

JC virus (JCV) is a human polyomavirus and the etiological agent of progressive multifocal leukoencephalopathy (PML), a fatal demyelinating disease of the white matter [1–3]. PML is primarily found in AIDS-patients, with between 3 % and 5 % of patients developing PML [4, 5]. However, PML has recently been diagnosed in patients undergoing immunomodulatory therapy with monoclonal antibodies against immune cells, such as Natalizumab, Rituximab, and Efalizumab, which indicates an immune component to the reactivation of the virus from latent reservoirs [6–9]. JCV is a non-enveloped polyomavirus with a genome comprised of 5 kb of double-stranded circular DNA with a bidirectional non-coding control region separating the early and late coding regions [10]. The early coding region of JCV encodes regulatory proteins, T-antigen, small t-antigen, and T’ splice variants expressed upon alternative splicing of the primary viral transcript [11]. The late coding region of JCV encodes the viral capsid proteins, VP1, VP2, and VP3, which are required for the formation of the viral capsid, as well as the small regulatory protein agnoprotein.

Previous studies have demonstrated that JCV undergoes cell-type specific activation, primarily in glial cells, which is proposed to be regulated at the transcriptional level [12]. Cellular proteins, such as SRSF1, play a major role in controlling JCV infection in glial cells [10]. We have recently identified alternative splicing factor SRSF1 as a negative regulator of JCV gene expression and replication in glial cells [10]. SRSF1 functions by targeting the JCV promoter and strongly inhibits JCV early and late gene transcription. Likewise, the down regulation of SRSF1 in astrocytes increases levels of viral gene expression and replication [10]. SRSF1 is a strong negative regulator of JCV gene expression as it suppresses both early and late gene transcription in glial cells [10, 13, 14]. Moreover, SRSF1 interaction with the CR3 region of JCV promoter sequences has been shown to be required for the ability of SRSF1 to regulate JCV gene expression and replication [14]. In order to proceed to a productive replication cycle, JC virus has to initiate the transcription of viral early and late genes by releasing the transcriptional silencing caused by host factors, such as SRSF1.

While cellular factors hold a negative pressure on viral gene expression in infected cells, viruses have evolved and developed strategies to bypass host defense mechanisms by utilizing mainly their regulatory proteins [15–22]. Here we investigated possible role of JCV regulatory proteins, T-antigen and agnoprotein, in regulation of viral transcriptional suppression mediated by SRSF1. Our results suggest that T-antigen but not agnoprotein is able to rescue and initiate viral transcription suppressed by SRSF1. These results have revealed a novel interaction between T-antigen and SRSF1 in controlling JCV gene transcription and replication, which may suggest a unique mechanism of JCV reactivation in patients who are at risk of developing PML.

## Results

### Effect of JCV regulatory proteins, T-antigen and agnoprotein, on SRSF1-mediated suppression of JCV transcription

**Fig. 1:**
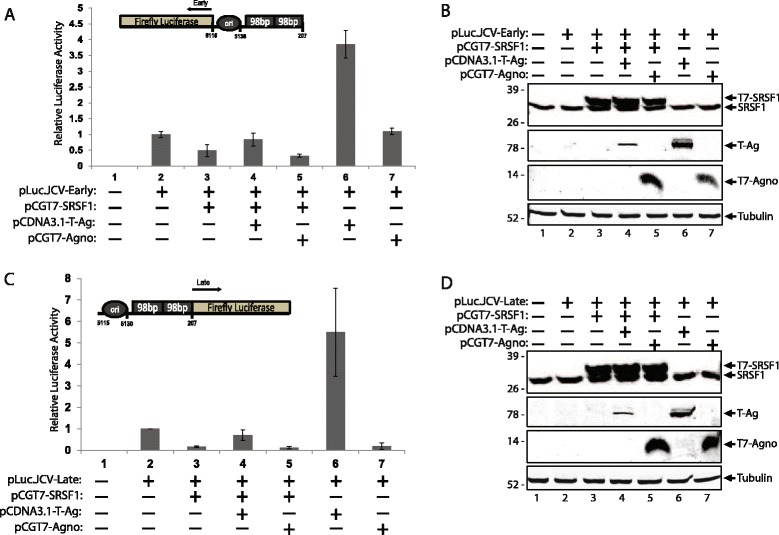
SRSF1 mediated suppression of JCV-early and -late gene transcription is complemented by T-Antigen. **a**. Luciferase assay showing the effects on transcriptional activity of the JCV early promoter region by SRSF1, T-antigen, and agnoprotein. PHFA cells were co-transfected with pLuc.JCV-Early and the listed expression plasmids. At 48 h post transfections, cell lysates were prepared and analyzed by luciferase reporter assay. Luminescence was determined and normalized to protein concentrations. Standard deviations were calculated from three independent experiments are presented as bar graph. Orientation of JCV Mad1 early promoter (GenBank: J02226.1) and firefly luciferase are schematized and shown in the upper panel of the bar graph. **b**. Western blot analysis of whole cell extracts prepared in parallel to the samples in panel A.SRSF1 encoded by plasmid with a T7 tag is distinguished from endogenous SRSF1 levels. **c**. PHFA cells were transiently transfected with pLuc.JCV-Late and listed plasmid combinations. At 48 h post transfections, cell lysates were prepared and analyzed by luciferase reporter assay. Luminescence was determined and normalized to protein concentrations. Standard deviations were calculated from three independent experiments are presented as bar graph. The orientation of JCV Mad1 late promoter (GenBank: J02226.1) and firefly luciferase are schematized and shown in the upper panel of the bar graph. **d**. Western blot analysis of whole cell extracts prepared in parallel to the samples in panel **c**

In order to investigate the role of JCV regulatory proteins T-antigen and agnoprotein in SRSF1-mediated suppression of JCV gene expression, primary human fetal astrocytes (PHFA) were transiently transfected with a luciferase reporter construct in both early and late orientations (pLuc.JCV-Early or pLuc.JCV-Late). In addition to transfection with the luciferase reporter construct, PHFA cells were co-transfected with plasmids encoding SRSF1 (pCGT7-SRSF1), T-antigen (pCDNA3.1-T-ag), and agnoprotein (pCGT7-agno) in varying combinations. At 48 h post transfections, JCV early and late transcription was analyzed by luciferase reporter assay as described in materials and methods. Luciferase assay of JCV-early gene transcription showed a strong suppression with SRSF1, which was rescued by co-expression of T-antigen, as well as a significant increase in transcription with the expression of T-antigen alone (Fig. 1a). However, there was no observed effect of agnoprotein on transcriptional suppression by SRSF1. Similarly, luciferase assay of JCV-late gene transcription yielded strong suppression of transcription with SRSF1, and a significant increase in transcription with T-antigen. When compared with the early gene transcription, SRSF1 possessed a much stronger suppression of late gene transcription (compare panel A, lane 3 with panel C, lane 3). Consistent with early gene transcription, T-antigen was also able to rescue the SRSF1-mediated transcriptional suppression of late promoter (Fig. 1c). Western blot analysis of the whole cell protein extracts were completed, demonstrating overexpression of SRSF1 and the expression of the viral proteins that were transfected in parallel to luciferase assays (Fig. 1b, d). These results suggest a novel role of viral T-antigen in suppression of cellular SRSF1 in glial cells.

### T-Antigen inhibits SRSF1 expression in glial cells

**Fig. 2:**
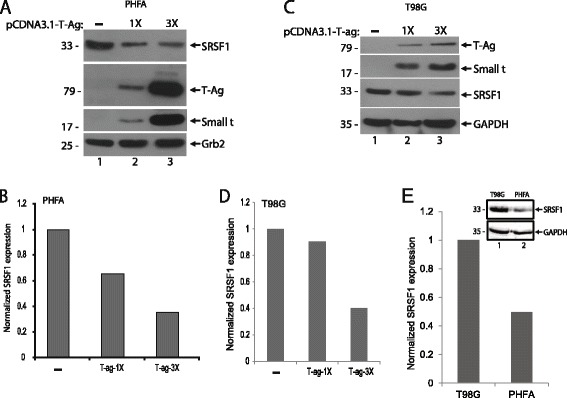
T-Antigen suppresses SRSF1 expression in glial cells. **a**. PHFA cells were transfected with increasing concentration of T-antigen expression plasmid. After 48 h post-transfection, whole cell protein extracts were collected. Western blot analyses were performed to detect levels of SRSF1, T-antigen, and small t antigen expressions. Grb2 served as a loading control. **b**. Bar graph representation of relative SRSF1 expression from panel **a**. Quantification of the intensity of the bands of SRSF1 from panel **a** were normalized to Grb2 band intensities and used to calculate relative expression. **c**. T98G cells were transfected with increasing concentration of T-antigen expression plasmid. After 48 h post-transfections, whole cell protein extracts were collected. Western blot analyses were performed to detect levels of SRSF1, T-antigen, and small t antigen expressions. GAPDH served as a loading control. **d**. Bar graph representation of relative SRSF1 expression from panel **c**. Quantification of the intensity of the bands of SRSF1 were normalized to GAPDH band intensities and used to calculate relative SRSF1 expression levels. **e**. Whole cell protein lysates from PHFA and T98G cells were prepared and analyzed by Western blotting for the detection of SRSF1 expression. GAPDH served as a loading control. Band intensity of the bands of SRSF1 were quantified, normalized to GAPDH, and shown as bar graph

To determine possible impact of T-antigen on cellular expression of SRSF1, both primary human fetal astrocytes (PHFA) and a human glioblastoma cell line (T98G) were transiently transfected with an expression vector encoding T-antigen in increasing concentrations. After 48 h post-transfection, cells were harvested and the whole cell protein extracts were analyzed by Western blotting for the detection of SRSF1 expression. As shown in Fig. 2a and b, expression of T-antigen caused a significant reduction in the basal expression levels of SRSF1 in PHFA cells in a dose dependent manner. On the other hand, consistent with primary glial cultures, expression of T-antigen also caused a significant reduction in SRSF1 levels in T98G cells (Fig. 2c and d). The early gene product small t antigen was also expressed by alternative splicing of T-antigen pre-mRNA and detected in the same immunoblots. These results suggest that JCV T-antigen can target and downregulate the expression of cellular SRSF1.

### T-Antigen targets SRSF1 promoter and inhibits its transcription

**Fig. 3:**
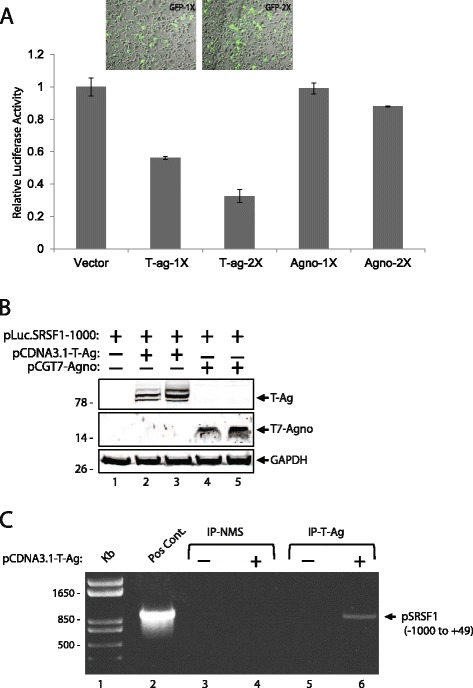
T-Antigen inhibits SRSF1 transcription through association with the promoter region. **a**. Effects of T-antigen and agnoprotein on SRSF1 transcription were tested by co-transfection studies. T98G cells were transiently transfected with pLuc.SRSF1(−1000) reporter plasmid and increasing concentrations of T-antigen, agnoprotein, and GFP expression plasmids. After 48 h, cell extracts were collected and analyzed for luminescence and normalized to protein concentrations. Relative luciferase activities were shown as bar graph. Standard deviations were calculated from three independent experiments. A representative panel of images of GFP expression from live cells transfected with increasing concentrations of pLEGFPC1 plasmid was also shown in the upper panel of the bar graph. **b**. Western blot analysis of whole cell extracts prepared in parallel to the samples in panel **a**. **c**. T-antigen is associated with SRSF1 promoter region. T98G cells were either transfected with T-antigen or left untransfected and proteins were cross-linked to DNA using formaldehyde, followed by ChIP analysis using antibody against T-antigen. Following immunoprecipitation, beads were eluted and underwent subsequent DNA purification. Purified DNA was analyzed by PCR for the −1000 to + 49 promoter region of SRSF1. In lane 2, the plasmid pLuc.SRSF1(−1000) was used as template and loaded as positive control. In lanes 5 and 6, samples from cells with and without T-antigen were subjected to immunoprecipitation by anti-T-Antigen antibody (IP-T-ag). In lanes 3 and 4, samples from cells with or without T-antigen expression were also subjected to immunoprecipitation by normal mouse serum (IP-NMS) as controls

In order investigate the molecular mechanism of T-antigen-mediated suppression of SRSF1 expression in glial cells, a luciferase assay reporting the transcriptional activity of the SRSF1 promoter was utilized. T98G cells were transiently transfected with a luciferase reporter construct consist of −1000 to +48 bp promoter region of SRSF1. Along with luciferase reporter construct, cells were co-transfected with plasmids encoding T-antigen or agnoprotein to determine the impact of these viral proteins on SRSF1 transcription. Cells were also transfected with an expression plasmid encoding green fluorescein protein (GFP) to access the transfection efficiencies. After 48 h, cells were harvested and processed for luciferase assay. It was observed that T-antigen, but not agnoprotein, suppressed the transcriptional activity of SRSF1 promoter (Fig 3a and b). Similar to our previous experiments (Fig. 2), it was found that higher levels of T-antigen correspond to a greater decrease in the transcriptional activity of SRSF1, which accounts for the decreased expression of SRSF1 in T-antigen expressing cells. To better understand the events associated with T-antigen suppression of SRSF1 promoter activity, we designed a series of experiments to assess the ability of T-antigen to bind to the SRSF1 promoter sequences. T98G cells were transfected with expression plasmid encoding T-antigen, cross-linked and possible interaction of T-antigen with SRSF1 promoter was analyzed by ChIP assay using antibodies to T-antigen as described in Materials and Methods. ChIP analysis of the cells demonstrated the association of T-antigen with SRSF1 promoter sequences (Fig. 3b).

## Discussion

Viral life cycles within the body are a fight between the ability of the host to recognize and quarantine or destroy the viruses and the virus’s ability to avoid this detection or avoid the mechanisms used to alter viral function. Therefore, the interplay between viral regulatory proteins and the host defense factors is a major regulator of the viral life cycle. While cellular factors holds a negative pressure on viral gene expression in infected cells, viruses have evolved and developed strategies to bypass host defense mechanisms by utilizing mainly their regulatory proteins [15–22]. One example by which a viral protein interacts with a host anti-viral protein is within the infection process of human cytomegalovirus (HCMV). When HCMV is detected by cells, an interferon gamma response is induced, resulting in the expression of multiple antiviral proteins, including the iron-sulfur cluster-binding antiviral protein viperin [15–17]. During the infection cycle, pre-expression of viperin results in inhibition of HCMV infection, however, the virus also is able to induce viperin expression independently of the IFN-dependent pathway [17, 18]. Although viperin contains antiviral activity, HCMV-induced viperin functions to disrupt cellular metabolism through localization to the mitochondria in infected cells, which results in enhancement of the HCMV infection process [18–22]. HCMV encodes a viral protein, viral mitochondrial inhibitor of apoptosis (vMIA), which interacts with viperin during HCMV infection, thus facilitating viral replication [18].

Similar to HCMV, JCV encodes viral regulatory proteins which are required for the progression of the lytic viral life cycle. We recently identified that cellular SRSF1 can overcome JCV reactivation by suppressing the expression of the viral genes [10, 13, 14]. Here, we investigated the molecular interplay between SRSF1 and JCV regulatory proteins T-antigen and agnoprotein in regulation of the viral transcription. Our results demonstrate that T-antigen is able to rescue the SRSF1-mediated transcriptional suppression seen in both early and late coding regions of the JCV genome. Likewise, it was found that T-antigen alone significantly increased viral transcription, however, a second viral regulatory protein, agnoprotein, had no effect on JCV transcription. To determine the mechanism behind T-antigen rescue of transcriptional suppression mediated by SRSF1, the impact of T-antigen expression on SRSF1 expression was analyzed. T-antigen expression decreased the expression of SRSF1, thus allowing for increased viral transcription. This step is hypothesized to be highly important within the JCV infection process, as T-antigen acts as a transcription factor for viral transcription and is able to autoregulate the viral promoter to drive the required viral processes for a productive infection cycle: including DNA replication and transcription of the late region allowing for agnoprotein and the capsid proteins to be expressed [23, 24]. The observed suppression of SRSF1 levels within cells expressing T-antigen was then demonstrated to be due to the T-antigen interaction with the SRSF1 promoter region leading to transcriptional inhibition.

## Conclusion

In summary, our results demonstrate a novel role of T-antigen in SRSF1-mediated suppression of JCV gene expression and provide basis for the further investigation of the role of the molecular interplay between cellular SRSF1 and viral T-antigen in JCV reactivation in glial cells leading to initiation of productive viral life cycle in patients who are at risk of developing PML.

## Materials and methods

### Cell lines and culture

The human glioblastoma multiforme cell line, T98G, was obtained from American Type Culture Collection (ATCC) and grown in Dulbecco’s Modified Eagle’s Medium (DMEM) containing 5 % heat-inactivated fetal bovine serum (FBS) and penicillin/streptomycin (100 ug/ml). T98G cells were maintained at 37 °C in a humidified environment with 5 % CO_2_. Primary human fetal astrocytes were cultured from the fetal human brain of a donor and provided by the Comprehensive NeuroAIDS Core facility at Temple University and cultured in Dulbecco’s Modified Eagle’s Medium/Nutrient Mixture F-12 (DMEM/F-12) containing 10 % heat-inactivated fetal bovine serum (FBS) penicillin/streptomycin (100 μg/ml), GlutaMax (100 μg/mL) and insulin (100 μg/mL). Cultured PHFA cells were maintained at 37 °C in a humidified atmosphere with 5 % CO_2_.

### Plasmid constructs

The T-antigen gene was cloned into the eukaryotic expression vector pcDNA3.1 (+) at the EcoRI restriction enzyme site and designated as pcDNA3.1-T-antigen previously [25]. pCGT7-SF2/ASF (SRSF1) expression plasmid was kindly provided by Javier F. Cáceres (Medical Research Council Human Genetics Unit, Western General Hospital, Edinburgh EH4 2XU, Scotland, United Kingdom) and was described previously [26]. The pCGT7-agno plasmid was described previously [27]. Briefly, agnoprotein coding sequence was cloned into the eukaryotic expression plasmid pCGT7 at Xba1/BamH1 restriction enzyme sites and designated as pCGT7-agno. The luciferase reporter construct pLuc-JCV-Early and pLuc-JCV-Late were made by blunt end cloning of the full-length Mad-1 NCCR into the SmaI site immediately upstream of the luciferase gene in the plasmid pGL3 (Promega, Madison WI) and described previously [28]. The luciferase reporter plasmid pLuc-SRSF1 was made by cloning the −1000 to +49 promoter region of SRSF1 gene into pGL3 vector at BamH1 site.

### Luciferase reporter assay

T98G cells were plated in 6-well tissue culture dishes and transiently transfected with pLuc-JCV-Early, pLuc-JCV-Late, or pLuc-SRSF1-1000 bp reporter plasmids in the presence or absence of expression plasmids for T-antigen and agnoprotein or pCDNA3.1 as control. At 48 h post-transfection, cells were extracted and lysed using reporter lysis buffer for the luciferase reporter system provided by the manufacturer Promega. After cell lysis, luciferase activity of samples was determined through the use of luciferase assay reagent (LAR). The luciferase activities were then corrected for protein concentrations and normalized to the basal levels of transcription, allowing for determination of the fold changes.

### Chromatin Immunoprecipitation Assay (ChIP)

T98G cells were transfected with pCDNA3.1-T-antigen. At 48 h post-transfection, proteins were cross-linked to DNA using formaldehyde at a final concentration of 1 %. After cross-linking, cells were lysed and subject to sonication to fragment the chromatin. After sonication, cells were incubated overnight with Protein G beads and anti-T antigen antibody (pAb-416, Calbiochem) for immunoprecipiation. After immunoprecipiation, the beads were washed and bound proteins were eluted using ChIP elution buffer (1 % SDS, 100 mM NaHCO_3_). After elution, cross-linking was reversed by using 5 M NaCl followed by Proteinase and RNase treatment. The sample was then purified for DNA using phenol/chloroform extraction followed by ethanol precipitation. Obtained DNA fragments were analyzed by PCR using primers; SRSF1-Promoter-Forward (−1000 to +47): 5’-ACCTTCCAAAGCTTTCCAGATTTCAG-3’ and SRSF1-Promoter-Reverse (+47 to +27): 5’-ACCTTCCACTCGAGGAAGGAAACAGC-3’. The PCR conditions were as follows: denaturing at 95° for 30 s, annealing at 58 °C for 40 s, and extension at 72 °C for 65 s. The PCR products were then resolved on a 1 % agarose gel.

### Western blot

Whole cell protein extracts were washed with PBS and lysed with TNN lysis buffer with protease inhibitors. Purified protein extracts were then heated to 95 °C for 5 min and resolved through sodium dodecyl sulfate polyacrylamide gel electrophoresis (SDS-PAGE). After resolution, the gel was transferred to a nitrocellulose membrane (Whatman, Germany) for three hours at 250 mÅ at 4 °C in transfer buffer (25 mM Tris pH 7.4, 200 mM glycine, 20 % methanol). After transfer, membranes were blocked for thirty minutes at room temperature with 5 % non-fat dry milk in 1X phosphate-buffered saline containing 0.1 % Tween-20 (PBST). After blocking, membranes were washed and incubated with primary antibodies at a 1:1000 dilution overnight at 4 °C. After primary antibody incubation, membranes were washed three times in PBST and incubated with secondary antibodies at a 1:5000 dilution at room temperature. Following secondary antibody incubation, membranes were visualized with the Odyssey CLx Imaging System (LI-COR). Primary antibodies used were anti-T-antigen (pAb-416, Calbiochem), anti-SRSF1 (ab12910, Abcam), anti-agnoprotein (Pab7903, house raised), anti-β-Tubulin (LI-COR), and anti-GAPDH (Cell Signaling Technology). Secondary antibodies used were IRDye 800CW goat anti-mouse (LI-COR) and IRDye 680RD goat anti-rabbit (LI-COR).
